# Supplementary material for: Synchronized proinsulin trafficking reveals delayed Golgi export accompanies β-cell secretory dysfunction in rodent models of hyperglycemia
Source: Sci Rep. 2023 Mar 30;13:5218. doi: 10.1038/s41598-023-32322-z (PMC10063606; doi:10.1038/s41598-023-32322-z)

Supplementary file

Synchronized proinsulin trafficking reveals delayed Golgi export accompanies β-cell secretory dysfunction in rodent models of hyperglycemia

Cierra K. Boyer [1,2], Casey J. Bauchle[2,3], Jianchao Zhang[4], Yanzhuang Wang[4,5] and

Samuel B. Stephens[2,3,6]

[1]Department of Neuroscience and Pharmacology, University of Iowa, Iowa City, IA, USA
[2]Fraternal Order of Eagles Diabetes Research Center, University of Iowa, Iowa City, IA, USA
[3]Department of Internal Medicine, Division of Endocrinology and Metabolism, University of Iowa, Iowa City, IA, USA
[4]Department of Molecular, Cellular, and Developmental Biology, University of Michigan, Ann Arbor, MI, USA
[5]Department of Neurology, School of Medicine, University of Michigan, Ann Arbor, MI, USA

[6]Corresponding author:
Samuel B. Stephens, Ph.D.
Fraternal Order of Eagles Diabetes Research Center
Department of Internal Medicine, Division of Endocrinology and Metabolism
University of Iowa
Iowa City, IA 52246, USA
Email: samuel-b-stephens@uiowa.edu
Tel. 319-335-4843

Supplemental figure legends

Supplemental Figure 1. Co-localization analysis of proCpepRUSH and SA. Mouse islets (C57BL6/J) treated with AdRIP-proCpepRUSH were examined 48 h post-infection. proCpepRUSH (green) localization was examined prior to biotin addition (A) or 3h post biotin (200 µM; B). Scale bar = 5 µm. Related to Figure 1.

Supplemental Figure 2. proCpepRUSH does not elicit the ER stress response. Isolated mouse islets were treated with AdRIP-proCpepRUSH, AdRIP-proCpepSNAP or no virus. 72 h post-infection, mRNA expression was examined and compared to islets treated for 18 h with thapsigargin (500 nM) as indicated (n=4-5 mice per group). Data represent the mean ± S.E.M. *$p < 0.05$ by 2 way-ANOVA with Sidak post-test analysis. Related to Figure 3.

Supplemental Figure 3. Insulin and proinsulin expression following dietary model of β-cell dysfunction. 8-10 week old male C57BL6/J mice were maintained on standard chow (SC) or Western diet for 8-10 weeks (n=3-5 mice per group). (**A**, **B**) mRNA expression was examined by qRT-PCR. Insulin (**C**) and proinsulin (**D**) content was determined from whole-cell lysates. (**E**) Ratio of proinsulin to insulin content. Data represent the mean ± S.E.M. * $p<0.05$ by Student t test. Related to Figure 4.

Supplemental Figure 4. Impaired insulin trafficking from the Golgi in a dietary model of β-cell dysfunction. 8-10 week old male C57BL6/J mice were maintained on standard chow

(SC) or Western diet for 8-10 weeks. (**A**) Golgi volume identified by GM130 immunostaining in proCpepRUSH positive β-cells was determined using Imaris (Bitplane) software (n=8 mice per group). (**B**-**C**) Isolated mouse islets were treated with AdRIP-proCpepRUSH. 48 h post-infection, islets were incubated at 19° C for 1 h and treated with biotin (200 μM) for an additional 2 h at 19° C to initiate proCpepRUSH (green) trafficking, but block Golgi exit. Islets were then shifted to 37° C for the indicated times to stimulate Golgi release. Cells were immunostained for GM130 (magenta) and counterstained with DAPI (blue). Islets were imaged by confocal microscopy. (**B**) Total proCpepRUSH-positive granule numbers were normalized to SC β-cells for each timepoint (n=5 mice per group; 7-18 cells per mouse). (**C**) Representative images are shown for Golgi release t = 2 h and 24 h. (**A**-**B**) Data represent the mean ± S.E.M. Not significant (ns) by Student t test. Related to Figure 5.

Movie S1. Time-lapse video of proCpepRUSH trafficking in primary β-cells. Mouse islets (C57BL6/J) treated with AdRIP-proCpepRUSH were examined 48 h post-infection. Biotin addition (200 μM) was used to initiate proCpepRUSH (green) trafficking. Image acquisition begins 12 min post-biotin treatment. Scale bar = 5 μm. Related to Figure 1.

Figure S1.

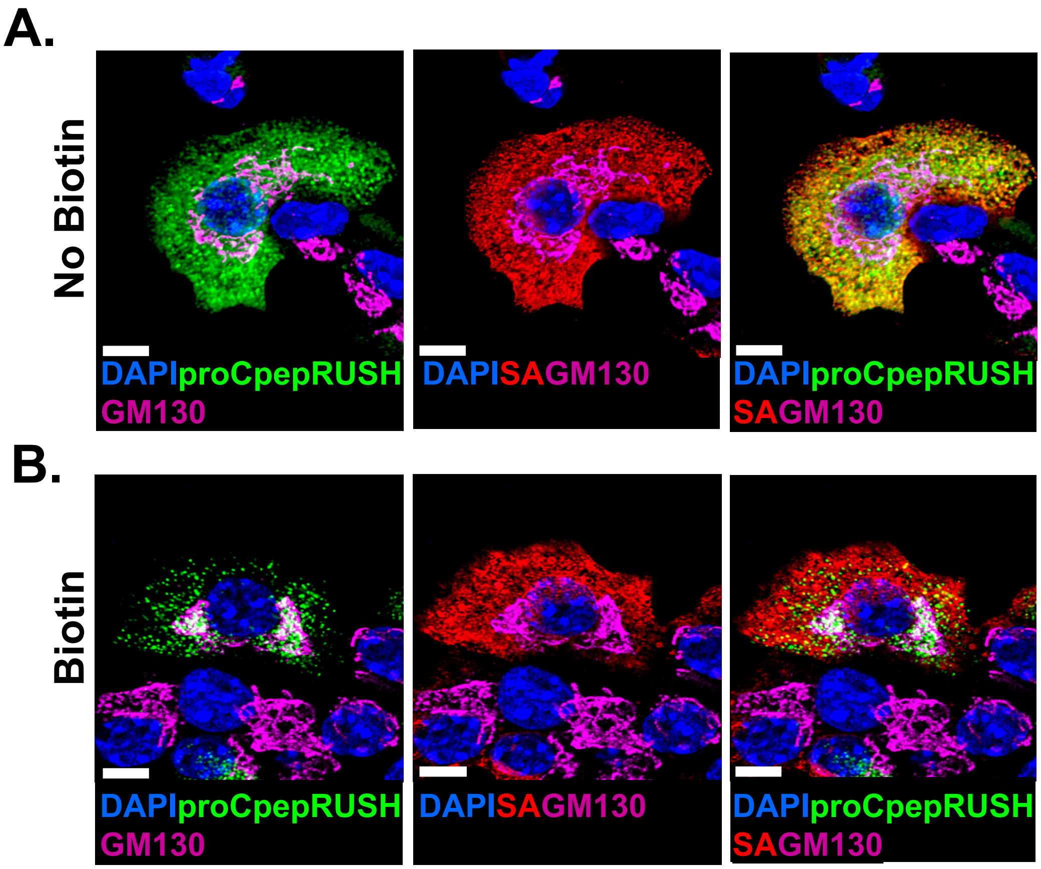
A.
No Biotin
DAPIproCpepRUSH
GM130
DAPISAGM130
DAPIproCpepRUSH
SAGM130
B.
Biotin
DAPIproCpepRUSH
GM130
DAPISAGM130
DAPIproCpepRUSH
SAGM130

Figure S2.

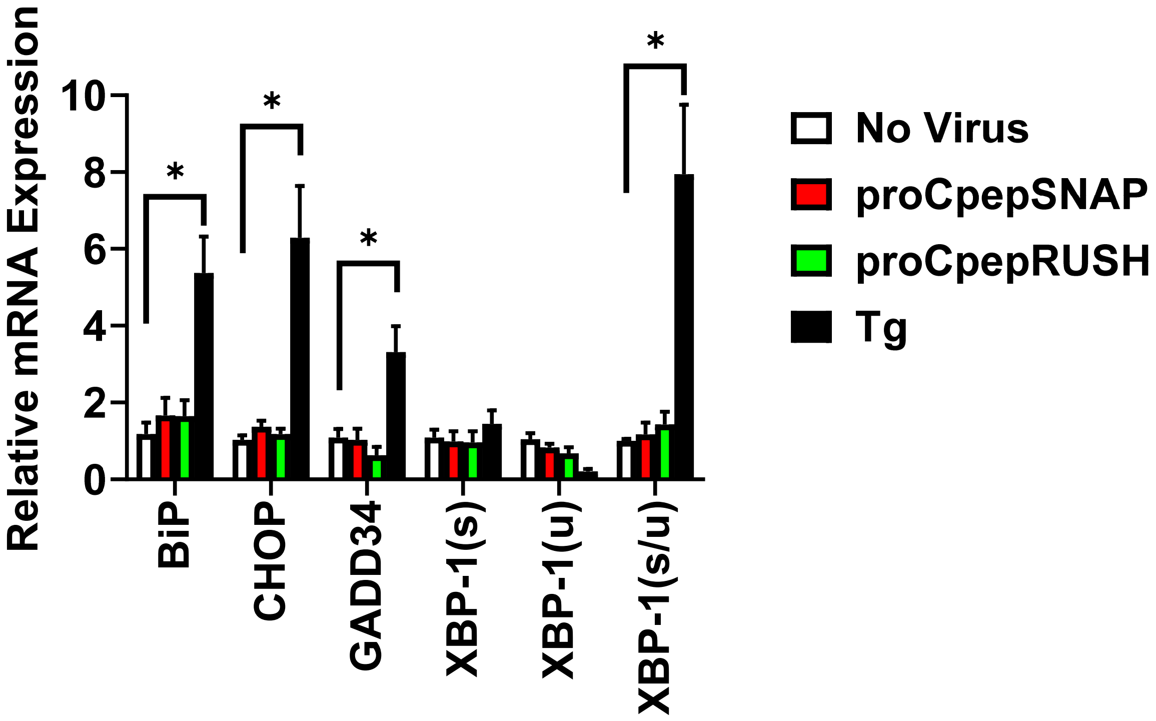
Relative mRNA Expression
10
8
6
4
2
0
*
*
*
*
BiP
CHOP
GADD34
XBP-1(s)
XBP-1(u)
XBP-1(s/u)
No Virus
proCpepSNAP
proCpepRUSH
Tg

Figure S3.

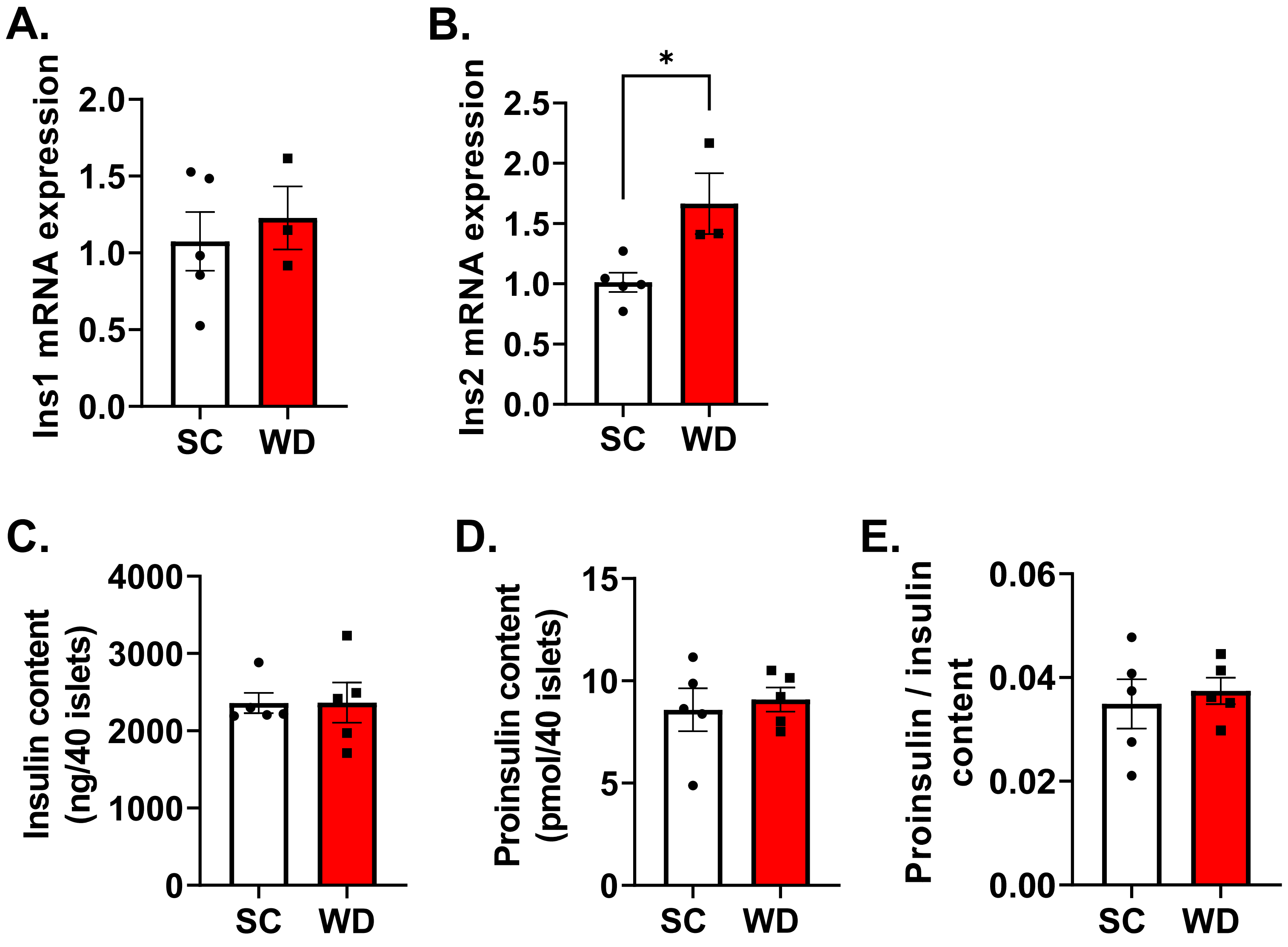

A.
Ins1 mRNA expression
2.0
1.5
1.0
0.5
0.0
SC
WD
B.
*
Ins2 mRNA expression
2.5
2.0
1.5
1.0
0.5
0.0
SC
WD
C.
Insulin content
(ng/40 islets)
4000
3000
2000
1000
0
SC
WD
D.
Proinsulin content
(pmol/40 islets)
15
10
5
0
SC
WD
E.
Proinsulin / insulin
content
0.06
0.04
0.02
0.00
SC
WD

Figure S4.

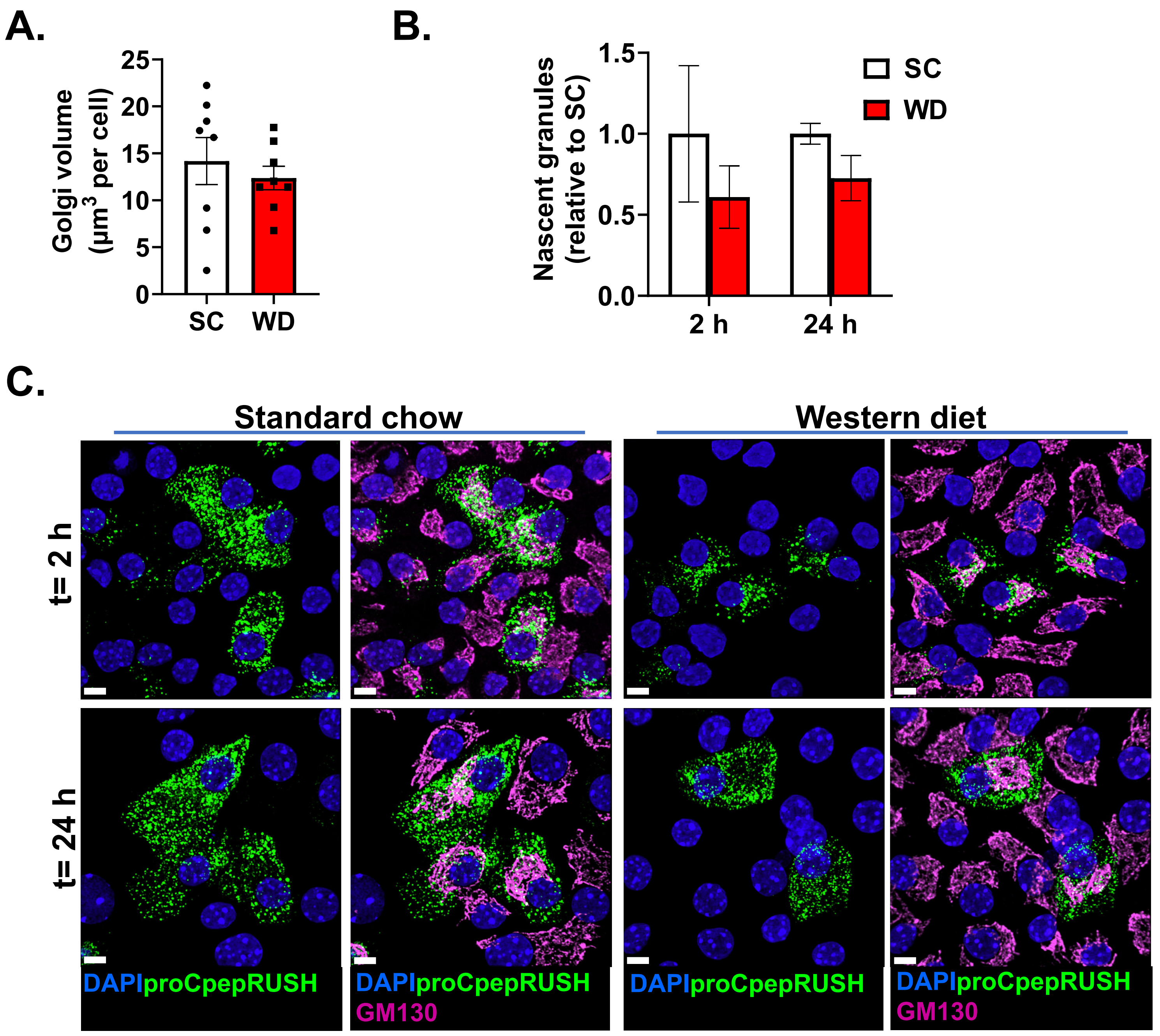
A.
Golgi volume (µm3 per cell)
25
20
15
10
5
0
SC
WD
B.
Nascent granules (relative to SC)
1.5
1.0
0.5
0.0
2 h
24 h
SC
WD
C.
Standard chow
Western diet
t= 2 h
t= 24 h
DAPIproCpepRUSH
DAPIproCpepRUSH GM130
DAPIproCpepRUSH
DAPIproCpepRUSH GM130

Uncropped immunoblots used in Figure 3A

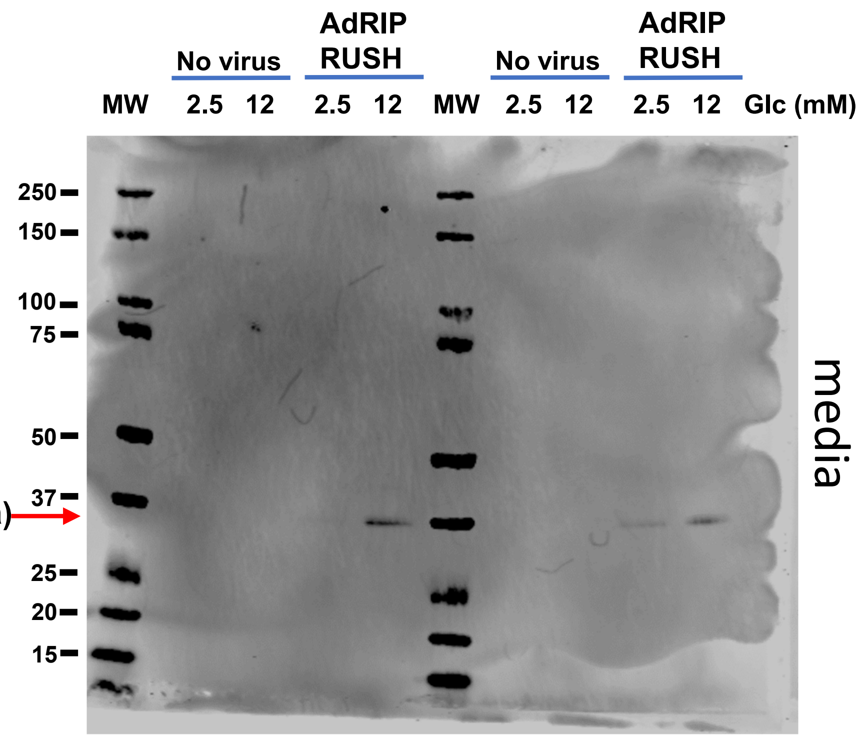

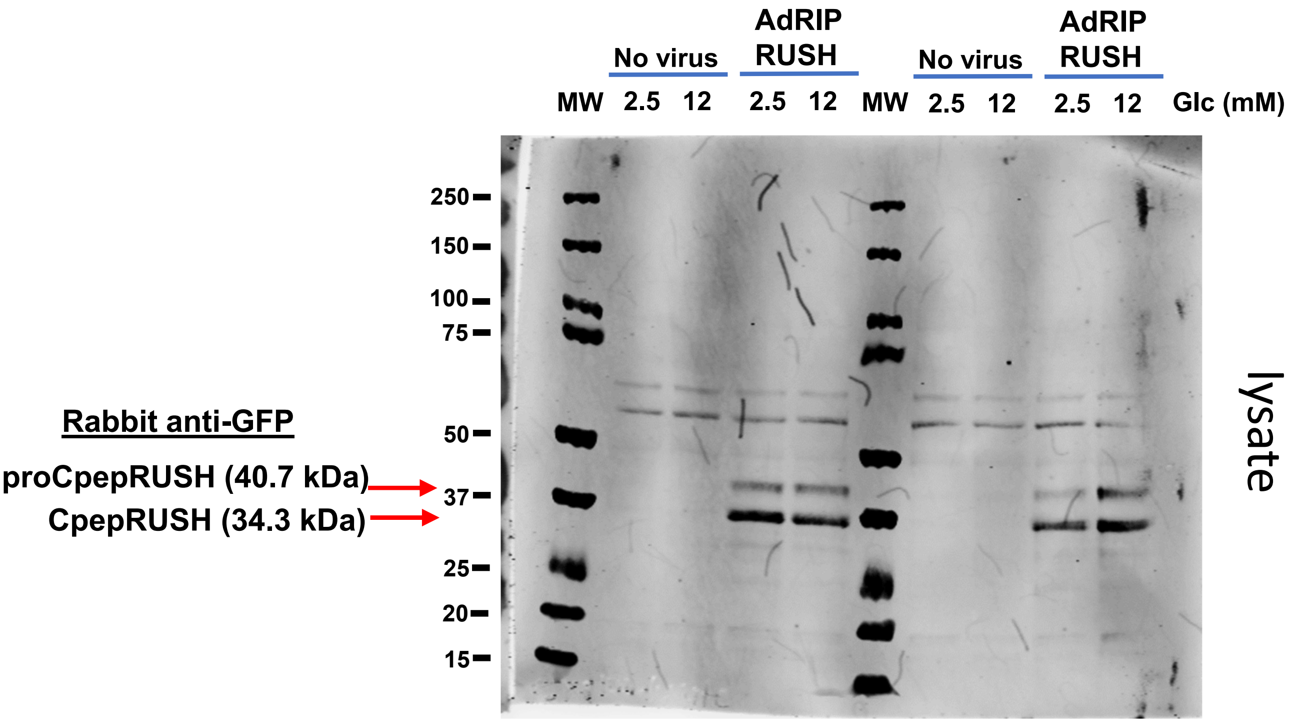

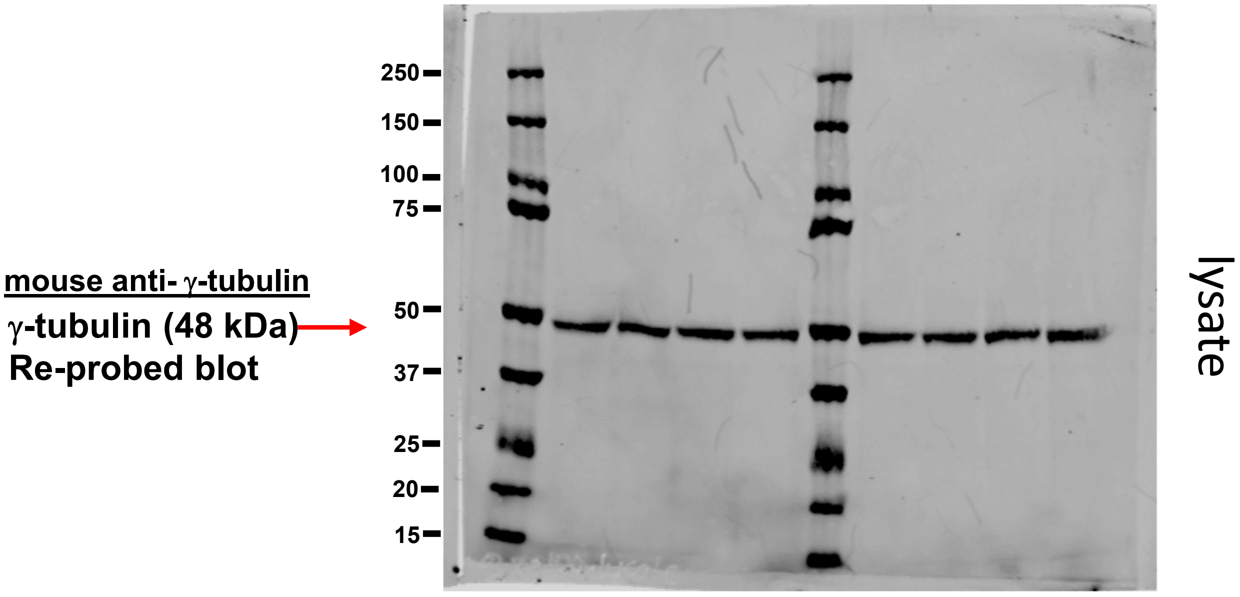

Uncropped immunoblots used in Figure 3D

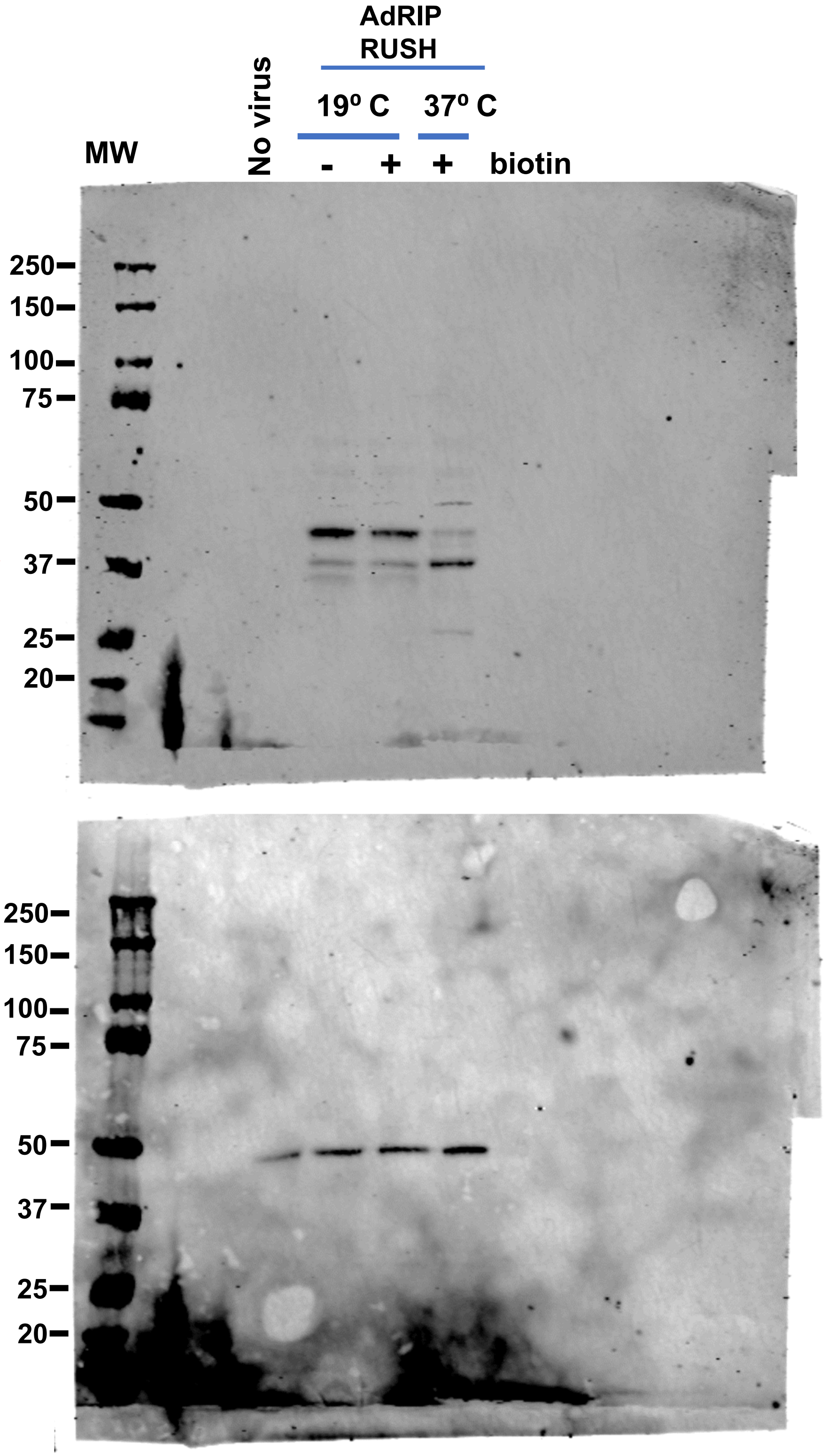

Supplement: Supplementary file 2 — Supplementary Information 1. [file 41598_2023_32322_MOESM2_ESM.pdf]
